# The Role of Teacher-Student Interpersonal Relations in Flipped Learning on Student Engagement

**DOI:** 10.3389/fpsyg.2021.741810

**Published:** 2021-08-12

**Authors:** Ruiguang Li

**Affiliations:** School of College English Teaching and Research, Henan University, Kaifeng, China

**Keywords:** flipped learning, students’ engagement, teacher-student interpersonal relations, interactive activity, student interactions

## Abstract

Education, in essence, is an interactive activity in which teacher and student interactions construct a learning path to raise knowledge. However, it is evident that this learning path is not merely cognitive. Thus, the role of interpersonal relationships should not be taken for granted. Teacher-student relationships are among the salient factors in effective teaching. Factors such as these trigger achievement, motivation, and engagement in students (Martin and Dowson, 2009), with student engagement in particular seeming like the keystone for educational achievement. One relative innovation that promotes student engagement and undertakes more effective learning and deeper knowledge of the materials is flipped learning (Kim, 2017). This theoretical review article was written to enlighten scholars, teachers, and learners with key concepts in interpersonal relations and their roles on student engagement in the context of flipped learning. In this study, some pedagogical implications were presented with the prospect of edifying the practice of teachers, students, and syllabus designers.

## Introduction

Student engagement is one of the contributing factors in the academic achievement of a learner and their intellectual evolution in education (Kahu and Nelson, 2017) and has been premeditated significantly by scholars throughout the world (Bond and Bedenlier, 2019; Bond et al., 2020). Student engagement relates to the vigor and power that students utilize in their learning community and is noticeable throughout every related scope of the behavioral, cognitive, or affective benchmarks. It is formed by a variety of physical and spiritual inspirations, containing the multifaceted interchange of relations, learning tasks, and settings of education (Bond et al., 2020).

Accordingly, it is noteworthy to scrutinize how student engagement can be promoted since there are several elements that manipulate student engagement. For instance, motivation boosts students in the learning process, with motivation being achieved from diverse sources such as parents, peers, teachers, and situations; nevertheless, in the domain of education, teachers have an eminent role that they must be held accountable for Martin (2014). Thus, this investigation of student engagement cannot forego mentioning the positive role of the teacher, as their performance is indeed a crucial aspect in the cultivation of student engagement (Groves et al., 2015). However, it should be noted that engagement is not an aspect of the student, but it can be relatively affected by circumstantial issues that are external to engagement (Sinclair et al., 2003). Concerning the manifestation of engagement, preserving positive teacher-student relationships as a type of contextual factor is the first step toward helping learners to be motivated, engaged, and thus become successful (Dotterer and Lowe, 2011). Undoubtedly, classrooms are considered intricate social organizations, and within them, teacher-student interactions are also multifaceted. Thus, the scope of the rapport between teachers and learners is essential in the mastery of student engagement (Pianta et al., 2012).

As put forward by Allen et al. (2013), the teacher-student association generates an emotional relation from the student, which causes student achievement. This is because the encouraging relationship between teacher and student develops collaboration and motivation in students, which are, in turn, tied to student success (Chen, 2016; Syahabuddin et al., 2020). Furthermore, the reciprocal care between teacher and learner may diminish undesirable emotions such as tedium, depression, and apprehension, and subsequently sustain student engagement (Furrer and Skinner, 2003).

Through positive relationships, students are encouraged to have autonomous voices to be able to talk over and express opinions spontaneously and safely about their thoughts and those of others, thus, being able to participate in social issues (Keating and Janmaat, 2015). The insight given by teachers should be open-minded, considerate, thoughtful, and free for discussion in order to motivate participation, not only in the classroom, but also outside it (Flanagan et al., 2007). Therefore, evaluating teacher-student relationships in the context of technology utilization is of the utmost consequence, as there is a bulk of inquiries on the role of technology in boosting student engagement (Chuang et al., 2018). Moreover, the majority of the previous studies have been focused on student engagement in different fields (Henrie et al., 2015; Derakhshan, 2021) and the significant role of teacher-student interaction assured the students’ success in the classroom (Li and Yang, 2021; Xie and Derakhshan, 2021).

As a type of blended learning, flipped learning has immensely burgeoned thanks to the continued pursuit for a method of learning that copes with the role of technology and supports the mutable desires of the new generation; subsequently, it has been embraced by teachers and scholars (Turan and Akdag-Cimen, 2020). Flipped learning is based on a constructivist agenda embedded in active learning (Webb and Doman, 2019), where the class is not only a place for better teacher-student relations but also for more peer communication (Wubbels et al., 2015). Through a high degree of interaction and collaborative learning practices in flipped learning, the problem-solving skills of learners are reinforced and their confidence is developed; thus, their success is ascertained (Yilmaz and Baydas, 2017; and Akçayır and Akçayır, 2018). The engagement of students in flipped learning has been proven in inquiries, as engaged students have been observed to be more involved in discussions, exert energy in classroom tasks, and demonstrate their enthusiasm to learn (Li et al., 2019; Li and Yang, 2021).

Despite the wide range of research undertaken to date on student engagement and the role of teacher-student interpersonal variables in flipped learning, one can notice the abundant research each concentrating on a particular construct. However, to the best of the knowledge of the researchers, the presentation of a review study on the above-mentioned issues in education and their connection with each other has been not taken into account. Based on this background, the present study embarked on reviewing the current issue.

## The Role of the Teacher in Student Engagement in Flipped Learning

With the arrival of positive psychology in the educational process, the role of emotion became dominant (MacIntyre and Mercer, 2014). D’Mello and Graesser (2012) evinced that positive emotions may bring about learning interest and motivation among learners, which subsequently result in student engagement. Student engagement lies within the learning procedure, and the role of teachers is crucial to illuminating several variances that exist between levels of classroom engagement (Hospel and Garland, 2016). As Shernoff et al. (2016) declared, the aptitude of teachers for forming the learning settings of their students is a process that affects student engagement. Indeed, it is the teacher who constructs classroom conditions, provides chances and possibilities to engage students (Collie et al., 2016), and creates a considerate and inspiring academic milieu (Shernoff et al., 2016).

Although flipped learning is primarily learner-centered (Bergmann and Sams, 2014), Moore et al. (2014) pinpointed that the most dominant attribute of the flipped classroom is its expansion of teacher-to-student and student-to-student communication and collaboration during class time. These relations are designated as the emotional ties with which students share that which can be interpersonal (Sabol and Pianta, 2012). One of the primary reasons flipped learning has been successful is the emergence of teacher-student interaction, as learners have become skilled enough to be involved in discussions (Kachka, 2012). Furthermore, through collaborative tasks in flipped learning within groups, opportunities are provided for students to communicate with their teacher; through this route, formative feedback can also be presented, and the relation between them is provoked (Yildiz Durak, 2018).

## Implications and Future Directions

The current review provides a venue for the role of interpersonal teacher-student relationships in the educational system. It is a significant supplement to the existing literature in understanding the role of these relationships in student engagement in the flipped classroom. Teachers might accentuate disengagement or low-level engagement students but as a panacea for this problem, the flipped classroom is assumed to contribute to deep learning, encourage them to be involved that sequentially results in the evolution of lifetime learning skills, as well (O’Flaherty et al., 2015). Teacher-student social relationships have also been anticipated as a defense alongside stress, emotional care in regular life, cooperation in shared tasks, and a foundation for progress (Martin, 2013). In the life of a learner, the energy obtained from teacher-student interpersonal relationships provides a vital route for motivation and engagement (Martin and Dowson, 2009).

In the flipped learning setting, employing teacher–learner communication could enrich student engagement and develop the transformation of the classroom and the learning accomplishment and success of students. By utilizing technology in the flipped classroom, thereby facilitating the communication between learners and teachers, the motivation of students is stimulated, and they subsequently turn into more active and engaged students. Through interaction and cooperation in the flipped classroom, the problem-solving capability of learners could also be fostered, along with their participation being strengthened and their confidence being enhanced (Fung et al., 2021).

The dependence of flipped learning on the growth of autonomy, empathy, and aptitude is supposed to increase student motivation (Abeysekera and Dawson, 2015). Regarding student engagement in the flipped classroom, students pinpointed that the use of classroom tasks triggered their critical thinking, making the tasks a worthwhile effort for learners and teachers (DeRuisseau, 2016). Considering the role of teacher-student relations as a key component of education (Xie and Derakhshan, 2021), the results can be taken for granted by syllabus designers to preserve venture in teaching students in the flipped learning approach through tasks that stimulate discussion.

Furthermore, engagement is aspectual and encompasses facets such as behavioral, emotional, and cognitive engagement, which can predominantly regulate the engagement utilizing tasks and activities of learners (Amiryousefi et al., 2019). Therefore, further research should look at how teachers understand and foster behavioral engagement and whether the formed tactics in flipped learning are being applied efficiently in terms of expanding the sense of belonging and emotional engagement of students. Concerning cognitive engagement, further research is also required to establish the mastery teachers have over the strategies used in flipped learning. Since the role of the teacher in forming and sustaining student engagement is critical, the present knowledge teachers have regarding student engagement must be re-constructed to determine whether teacher perceptions might affect existing engagement practices.

However, this review displayed that there are still lacunas in the literature that may reflect what teachers truly do in flipped classrooms to cultivate student engagement and what they can do to promote and support student engagement. In this manner, teachers and future teachers can discern how to relate the flipped learning model to their own routines in order to generate the learner-centric learning atmospheres that have been underlined by constructivists. Since the student-teacher relationship is significant in classes, more studies should be implemented to emphasize the learning styles and personality types of teachers and how these influence the way materials are suggested, adapted, and implemented in flipped learning. Finally, as a fertile area of research, more research could investigate the ways that teachers attempt to guarantee student responsibility during flipped learning progress. Based on the literature, it is challenging to envisage the extent to which teachers can promote student engagement based on their interpersonal relations, so the process of their teaching should be further investigated.

## Author Contributions

The author confirms being the sole contributor of this work and has approved it for publication.

## Conflict of Interest

The author declares that the research was conducted in the absence of any commercial or financial relationships that could be construed as a potential conflict of interest.

## Publisher’s Note

All claims expressed in this article are solely those of the authors and do not necessarily represent those of their affiliated organizations, or those of the publisher, the editors and the reviewers. Any product that may be evaluated in this article, or claim that may be made by its manufacturer, is not guaranteed or endorsed by the publisher.

## References

[B1] Abeysekera L. Dawson P. (2015). Motivation and cognitive load in the flipped classroom: definition, rationale and a call for research. *High. Educ. Res. Dev.* 34 1–14. 10.1080/07294360

[B2] Akçayır G. Akçayır M. (2018). The flipped classroom: a review of its advantages and challenges. *Comput. Educ.* 126 334–345. 10.1016/j.compedu.2018.07.021

[B3] Allen E. Kruger C. Leung F. Y. Stephens J. C. (2013). Diverse perceptions of stakeholder engagement within an environmental modeling research team. *J. Environ. Stud. Sci.* 3 343–356. 10.1007/s13412-013-0136-x

[B4] Amiryousefi M. Amirian Z. Ansari A. (2019). Relationship between classroom environment, teacher behavior, cognitive and emotional engagement, and state motivation. *J. English Lang. Teach. Learn.* 11 27–56.

[B5] Bergmann J. Sams A. (2014). Flipping for mastery. *Educ. Leadersh.* 71 24–29.

[B6] Bond M. Bedenlier S. (2019). Facilitating student engagement through educational technology: towards a conceptual framework. *J. Interact. Media Educ.* 11 1–14.

[B7] Bond M. Buntins K. Bedenlier S. Zawacki-Richter O. Kerres M. (2020). Mapping research in student engagement and educational technology in higher education: a systematic evidence map. *Int. J. Educ. Technol. High. Educ.* 17 1–30. 10.1007/978-3-319-46034-5_1

[B8] Chen J. (2016). Understanding teacher emotions: the development of a teacher emotion inventory. *Teach. Teach. Educ.* 55 68–77. 10.1016/j.tate.2016.01.001

[B9] Chuang H. H. Weng C. Y. Chen C. H. (2018). Which students benefit most from a flipped classroom approach to language learning? *Br. J. Educ. Technol.* 49 56–68. 10.1111/bjet.12530

[B10] Collie R. J. Martin A. J. Curwood J. S. (2016). Multidimensional motivation and engagement for writing: construct validation with a sample of boys. *Educ. Psychol.* 36 771–791. 10.1080/01443410.2015.1093607

[B11] Derakhshan A. (2021). The predictability of Turkman students’ academic engagement through Persian language teachers’ nonverbal immediacy and credibility. *Journal of Teach. Persian Speakers Lang.* 10 3–26.

[B12] DeRuisseau L. R. (2016). The flipped classroom allows for more class time devoted to critical thinking. *Adv. Physiol. Educ.* 40 522–528. 10.1152/advan.00033.2016 28145270

[B13] D’Mello S. Graesser A. (2012). “Emotions during learning with auto-tutor,” in *Adaptive Technologies For Training And Education*, eds Durlach P. Lesgold A. (Cambridge: Cambridge University Press), 117–139. 10.1017/CBO9781139049580.010

[B14] Dotterer A. M. Lowe K. (2011). Classroom context, school engagement, and academic achievement in early adolescence. *J. Youth Adolesc.* 40 1649–1660. 10.1007/s10964-011-9647-5 21400208

[B15] Flanagan C. A. Cumsille P. Gill S. Gallay L. S. (2007). School and community climates and civic commitments: patterns for ethnic minority and majority students. *J. Educ. Psychol.* 99 421–431. 10.1037/0022-0663.99.2.421

[B16] Fung C. H. Besser M. Poon K. K. (2021). Systematic literature review of flipped classroom in Mathematics. *Eur. J. Math. Sci. Technol. Educ.* 17 1–17. 10.29333/ejmste/10900

[B17] Furrer C. Skinner E. A. (2003). Sense of relatedness as a factor in children’s aca-demic engagement and performance. *J. Educ. Psychol.* 95 148–162. 10.1037/0022-0663.95.1.148

[B18] Groves M. Sellars C. Smith J. Barber A. (2015). Factors affecting student engagement: a case study examining two cohorts of students attending a post -1992 university in the United Kingdom. *Int. J. High. Educ.* 4 27–37. 10.5430/ijhe.v4np27

[B19] Henrie C. R. Halverson L. R. Graham C. R. (2015). Measuring student engagement in technology-mediated learning: a review. *Comput. Educ.* 90 36–53. 10.1016/j.compedu.2015.09.005

[B20] Hospel V. Garland B. (2016). Are both classroom autonomy and structure equally important for students’ engagement? a multilevel analysis. *Learn. Instruct.* 41, 1–10. 10.1016/j.learninstruc.2015.09.001

[B21] Kachka P. (2012). *Understanding the Flipped Classroom: Part 2. Faculty Focus.* Available online at: https://www.facultyfocus.com/articles/blended-flipped-learning/understanding-the-flippedclassroom-part-2/ (accessed May 15, 2021).

[B22] Kahu E. R. Nelson K. (2017). Student engagement in the educational interface: understanding the mechanisms of student success. *High. Educ. Res. Dev.* 37 58–71. 10.1080/07294360.2017.1344197

[B23] Keating A. Janmaat J. G. (2015). Education through citizenship at school: do school activities have a lasting impact on youth political engagement. *Parliam. Aff.* 69, 409–429. 10.1093/pa/gsv017

[B24] Kim J. Y. (2017). A study of students’ perspectives on a flipped learning model and associations among personality, learning styles and satisfaction. *Innov. Educ. Teach. Int.* 55 314–324. 10.1080/14703297.2017.1286998

[B25] Li L. Yang S. (2021). Exploring the influence of teacher-student interaction on university students’ self-efficacy in the flipped classroom. *J. Educ. Learn.* 10 84–90. 10.5539/jel.v10n2p84

[B26] Li S. Jiao F. Zhang Y. Xu X. (2019). Problems and changes in digital libraries in the age of big data from the perspective of user services. *J. Acad. Librariansh.* 45 22–30. 10.1016/j.acalib.2018.11.012

[B27] MacIntyre P. D. Mercer S. (2014). Introducing positive psychology to SLA. *Stud. Second Lang. Learn. Teach.* 4 153–172. 10.14746/ssllt.2014.4.2.2

[B28] Martin A. J. (2013). Improving the achievement, motivation, and engagement of students with ADHD: the role of personal best goals and other growth-based approaches. *J. Psychol. Couns. Sch.* 23 143–155. 10.1017/jgc.2013.4

[B29] Martin A. J. (2014). “Interpersonal relationships and students’ academic and non-academic development: what outcomes peers, parents, and teachers do and do not impact,” in *Interpersonal Relationships*, eds Tartwijk J. Zandvliet D. Mainhard T. den Brok P. (London: Sense).

[B30] Martin A. J. Dowson M. (2009). Interpersonal relationships, motivation, engagement, and achievement: yields for theory, current issues, and educational practice. *Rev. Educ. Res.* 79 327–365. 10.3102/0034654308325583

[B31] Moore A. J. Gillett M. R. Steele M. D. (2014). Fostering student engagement with the flip. *Mathe. Teach.* 107 420–425. 10.5951/mathteacher.107.6.0420

[B32] O’Flaherty J. Phillips C. Karanicolas S. Snelling C. Winning T. (2015). Corrigendum to the use of flipped classrooms in higher education: a scoping review. *Int. High. Educ.* 25 85–95. 10.1016/j.iheduc.2015.02.002

[B33] Pianta R. C. Hamre B. K. Allen J. P. (2012). “Teacher-student relationships and engagement: conceptualizing, measuring, and improving the capacity of classroom interactions,” in *Handbook Of Research On Student Engagement*, eds Christenson S. L. Reschly A. L. Wylie C. (Boston, MA: Springer), 365–386. 10.1007/978-1-4614-2018-7_17

[B34] Sabol T. J. Pianta R. C. (2012). Recent trends in research on teacher–child relationships. *Attach. Hum. Dev.* 14 213–231. 10.1080/14616734.2012.672262 22537521

[B35] Shernoff D. J. Kelly S. Tonks S. M. Anderson B. Cavanagh R. F. Sinha S. (2016). Student engagement as a function of environmental complexity in high school classrooms. *Learn. Instr.* 43 52–60. 10.1016/j.learninstruc.2015.12.003

[B36] Sinclair M. F. Christenson S. L. Lehr C. A. Anderson A. R. (2003). Facilitating student engagement: lessons learned from check & connect longitudinal studies. *Calif. Sch. Psychol.* 8 29–41. 10.1007/bf03340894

[B37] Syahabuddin K. Fhonna R. Maghfirah U. (2020). Teacher-student relationships: an influence on the English teaching-learning process. *Stud. English Lang. Educ.* 7 438–451. 10.24815/siele.v7i2.16922

[B38] Turan Z. Akdag-Cimen B. (2020). Flipped classroom in English language teaching: a systematic review. *Comput. Assist. Lang. Learn.* 33 590–606. 10.1080/09588221.2019.1584117

[B39] Webb M. Doman E. (2019). Impacts of flipped classrooms on learner attitudes towards technology-enhanced language learning. *Comput. Assist. Lang. Learn.* 33 240–274. 10.1080/09588221.2018.1557692

[B40] Wubbels T. Brekelmans M. den Brok P. Wijsman L. Mainhard T. van Tartwijk J. (2015). “Teacher-student relationships and classroom management,” in *Handbook of Classroom Management*, eds Emmer E. T. Sabornie E. J. (New York, NY: Routledge), 363–386.

[B41] Xie F. Derakhshan A. (2021). A conceptual review of positive teacher interpersonal communication behaviors in the instructional context. *Front. Psychol.* 12:2623. 10.3389/fpsyg.2021.708490 34335424 PMC8319622

[B42] Yildiz Durak H. (2018). Flipped learning readiness in teaching programming in middle schools: modelling its relation to various variables. *J. Comput. Assist. Learn.* 34 939–959. 10.1111/jcal.12302

[B43] Yilmaz R. M. Baydas O. (2017). An examination of undergraduates’ metacognitive strategies in pre-class asynchronous activity in a flipped classroom. *Educ. Technol. Res. Dev.* 65 1547–1567. 10.1007/s11423-017-9534-1

